# AIDS-related primary central nervous system lymphoma: a Norwegian national survey 1989–2003

**DOI:** 10.1186/1471-2407-8-225

**Published:** 2008-08-06

**Authors:** Ingfrid S Haldorsen, Jostein Kråkenes, Anne K Goplen, Oona Dunlop, Olav Mella, Ansgar Espeland

**Affiliations:** 1 Department of Radiology, Haukeland University Hospital, Bergen, Norway; 2 Section for Radiology, Department of Surgical Sciences, University of Bergen, Bergen, Norway; 3 Department of Pathology, Ullevaal University Hospital, Oslo, Norway; 4 Department of Infectious Diseases and Department of Acute Medicine, Ullevaal University Hospital, Oslo, Norway; 5 Department of Oncology and Medical Physics, Haukeland University Hospital, Bergen, Norway; 6 Section of Oncology, Institute of Medicine, University of Bergen, Bergen, Norway

## Abstract

**Background:**

Primary central nervous system lymphoma (PCNSL) is a frequent complication in acquired immunodeficiency syndrome (AIDS). The objective of this survey was to investigate incidence, clinical features, radiological findings, histologic diagnosis, treatment and outcome for all patients with histologically verified AIDS-related PCNSL diagnosed in Norway in 1989–2003.

**Methods:**

We identified the patients by chart review of all cases recorded as PCNSL in The Norwegian Cancer Registry (by law recording all cases of cancer in Norway) and all cases recorded as AIDS-related PCNSL in the autopsy registry at a hospital having 67% autopsy rate and treating 59% of AIDS patients in Norway, from 1989 to 2003. Histologic material and radiological images were reviewed. We used person-time techniques to calculate incidence rates of PCNSL among AIDS patients based on recordings on AIDS at the Norwegian Surveillance System for Communicable Diseases (by law recording all cases of AIDS in Norway).

**Results:**

Twenty-nine patients had histologically confirmed, newly diagnosed AIDS-related PCNSL in Norway from 1989–2003. Only 2 patients had this diagnosis established while alive. AIDS patients had 5.5% lifetime risk of PCNSL. Their absolute incidence rate of PCNSL per 100 person-years was 1.7 (95%CI: 1.1–2.4) and decreased during the consecutive 5-year periods from 3.6, to 2.5, and to 0.4 (p < 0.001). Median survival from initial symptom of PCNSL was 2.3 months, but one patient was still alive 4 years after completed radiotherapy.

**Conclusion:**

This is the first national survey to confirm decreasing incidence of AIDS-related PCNSL. Despite dismal survival in most patients, the possibility of long term survival should prompt more aggressive diagnostics in suspected PCNSL.

## Background

Primary central nervous system lymphoma (PCNSL) has been diagnosed in at least 2% of individuals infected with human immunodeficiency virus (HIV) [1], and in 9–14% of acquired immunodeficiency syndrome (AIDS)-autopsies [2-4]. PCNSL is, after toxoplasmosis, the most common cause of focal brain lesions in AIDS patients [1,5]. The reported median survival in AIDS-related PCNSL is 1.0–2.5 months with supportive care only [6,7] and 2.0–5.5 months with whole brain radiation therapy [1,8]. The incidence of PCNSL among AIDS patients has been reported to decline in the era of highly active antiretroviral therapy (HAART), introduced in 1996 [8-14].

Few studies on AIDS related PCNSL were population based [3,9,15-17]. This survey is, to the best of our knowledge, the first comprising a whole country. Its objective was to investigate incidence, clinical features, radiological findings, histologic diagnosis, treatment and outcome for all patients with histologically verified AIDS-related PCNSL diagnosed in Norway in 1989–2003.

## Methods

This study complied with the Helsinki Declaration. The Regional Committee for Medical Research Ethics, the Directorate for Health and Social Affairs and The Data Inspectorate approved the study and waived informed consent.

Norway (population of 4.2 million in 1989 and 4.6 million in 2003) has mandatory cancer reporting to the Norwegian Cancer Registry. This national registry recorded 180 patients with PCNSL diagnosed from 1 January 1989 to 31 December 2003. Medical records of all patients were reviewed. Histologically verified AIDS-related PCNSL was found in 23 of the 180 patients (98 had non-AIDS PCNSL and 59 no PCNSL) [18]. During the studied 15-year period Ullevaal University Hospital treated 59% (423/723) of reported new cases of AIDS in Norway and autopsied 67% (186/278) of their AIDS deaths. A search in their AIDS autopsy registry identified 6 additional cases of AIDS-related PCNSL.

This study sample thus consisted of 29 AIDS patients with histologically verified PCNSL; 27 were diagnosed and treated at Ullevaal University Hospital. Clinical features, histologic diagnosis, radiological findings, treatment and outcome were registered. HAART was defined as the administration of three or more antiretroviral agents including a protease inhibitor or non-nucleoside reverse transcriptase inhibitor. One pathologist (AKG) re-examined histologic material of all these patients from biopsy (n = 2) or autopsy (n = 27) and reclassified it according to the current WHO-classification (2001). For immunohistochemistry, the antibodies CD3, CD20 and Ki67 were used as a minimum. Epstein-Barr virus hybridization was performed in 25 patients. Twenty-six patients had a complete autopsy of the entire body (excluding systemic lymphoma). The remaining 3 patients underwent biopsy (n = 2) or autopsy (n = 1) of the brain only; none of them had signs of lymphoma at chest- and abdomen imaging (n = 3) or bone marrow biopsy (n = 2). Cause of death was established from medical records, autopsy reports and death certificates.

In 14 included patients (1 of 12 diagnosed in 1989–1993, 13 of 17 diagnosed in 1994–2003) images from computed tomography (CT) (in 14) and magnetic resonance imaging (MRI) (in 4) prior to histologic diagnosis were available for review. (In Norway hospitals are not obliged to store radiological images for more than 10 years.) A neuroradiologist (JK) and a general radiologist (ISH) reviewed the images, first individually and then together. Results are based on consensus from the joint review.

Number of new cases with AIDS and number of AIDS deaths in Norway each year were obtained from MSIS (The Norwegian Surveillance System for Communicable Diseases). Named AIDS reporting from laboratories and clinicians to this national surveillance system has been mandatory in Norway since 1983 [19].

### Statistical analyses

We calculated absolute incidence rates of PCNSL among persons with AIDS based on numbers of newly diagnosed AIDS-related PCNSL and numbers of patients living with AIDS the same year (PYAR; Person-Years-At-Risk) (data from MSIS). Trend test and 95% confidence interval (CI) for incidence was calculated according to the Poisson distribution.

Duration of survival was calculated as time between date of first symptom attributable to PCNSL and date of death or January 1^st ^2008. Survival curves were calculated and plotted using the method of Kaplan and Meier. All p-values in the analyses are two-sided. The analyses were performed with Stata 9.0 (College Station, TX) and SPSS 14.0 (Chicago, IL).

## Results

**Figure 1:**
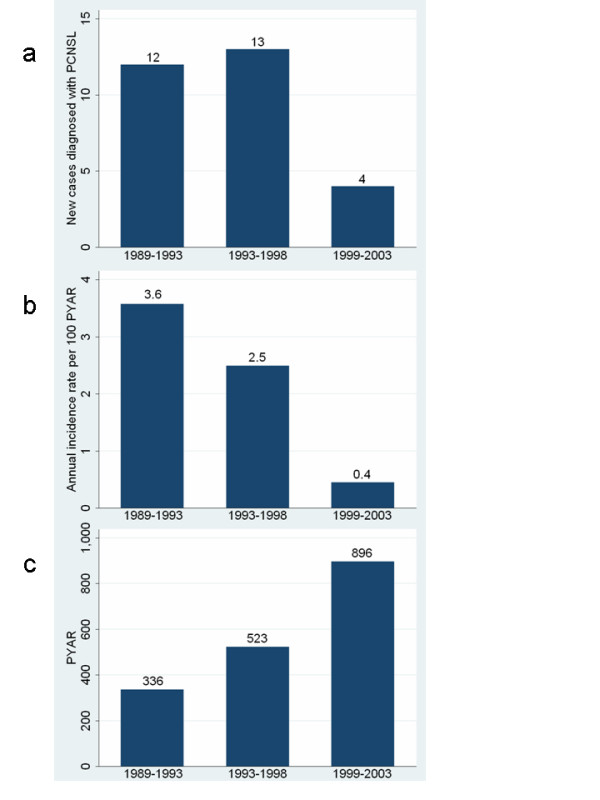
**AIDS-related PCNSL in Norway: new cases, incidence rates and Person-Years-At-Risk (PYAR)**. New cases diagnosed with AIDS-related PCNSL (a), annual incidence rate per 100 Person-Years-At-Risk (PYAR) (b) and PYAR (c) the three 5-year periods: 1989–1993, 1994–1998 and 1999–2003, in Norway.

In Norway the absolute incidence rate of PCNSL among AIDS patients per 100 person-years in 1989–2003 was 1.7 (95%CI: 1.1–2.4) and decreased from 3.6, to 2.5, and to 0.4 the consecutive 5-year periods (p < 0.001) (Figure 1). The average annual incidence rate of AIDS-related PCNSL among inhabitants in Norway was 0.44 per million (95%CI: 0.30–0.64). The proportion of AIDS-deaths diagnosed with concomitant PCNSL was 5.5% (28/508). This proportion was stable over time (5.3% (12/227), 6.0% (13/215) and 4.6% (3/66) in 1989–1993, 1994–1998 and 1999–2003, respectively; Fischer's Exact Test: p = 0.96).

Patient characteristics are listed in Table 1. Twenty-six (90%) of the 29 patients were male. Mean age at diagnosis of PCNSL was 39.0 years. The histologic diagnosis was diffuse large B-cell lymphoma in all patients. Only 2 patients had this diagnosis established while alive (in 2000 and 2003, based on open biopsy). Immunohistochemistry was positive with CD20 and Ki67 and negative with CD3 in 29/29 (100%). Epstein-Barr virus hybridization was positive in 25/25 (100%). Polymerase chain reaction (PCR) of the cerebrospinal fluid (CSF) performed prior to death demonstrated positive Epstein-Barr virus DNA in 3/5 (60%) and negative Toxoplasma gondii DNA in 2/2 (100%).

**Table 1:** Characteristics of AIDS patients with PCNSL in Norway during 1989–2003 (n = 29).

	n %
Gender	
Female	3 (10)
Male	26 (90)
Supposed mode of transmission	
Homosexual	17 (59)
Heterosexual	8 (28)
Drug addict	2 (7)
Unknown	2 (7)
Duration of HIV-infection prior to AIDS: mean; median (range) months	38.6; 32.0 (0–118)
AIDS-defining disease	
Pneumocystis jirovecii pneumonia	16 (55)
PCNSL	6 (21)
Tuberculosis	1 (3)
Cytomegalovirus infection	1 (3)
Kaposis sarcoma	2 (7)
Candida esophagitis	3 (10)
Duration of AIDS prior to PCNSL: mean; median (range) months	19.1; 15.0 (0–81)
Age at diagnosis of PCNSL: mean; median (range) years	39.0; 37.9 (24–54)
Symptoms on admission	
Focal neurological	11 (38)
Personality change	14 (48)
Cerebellar symptoms/vertigo	10 (35)
Headache	10 (35)
Visual disturbance	6 (21)
Epilepsy	8 (28)
Performance status on admission	
WHO PS 0–1	8 (28)
WHO PS 2–4	21 (72)
CD4 lymphocyte count (cells/μL): mean; median (range) (n = 24)^a^	26.6; 10.0 (0–190)
Lesions on CT/MR according to imaging report	
No focal lesion	4 (14)
Single	8 (28)
Multiple	17 (59)
Lesions on CT/MR at review (in 14 patients)	
Mean; median (range) number of lesions per patient	2.6; 2.0 (0–7)
Supratentorial involvement	14 (100)
Lobes	11 (79)
Deep structures	8 (57)
Posterior fossa involvement	2 (14)
Mean; median (range) size (diameter in mm) of lesions (n = 37)	24.2; 23.0 (6–55)
Treatment received	
Steroid medication	11 (38)
Radiotherapy^b^	2 (7)
HAART	4 (14)
Toxoplasma therapy^c^	20 (69)

PCNSL = primary central nervous system lymphoma; WHO PS = World Health Organization performance status; CT = computed tomography; MRI = magnetic resonance imaging; HAART = highly active antiretroviral therapy.

^a^CD4 lymphocyte count at time of first symptom attributable to PCNSL.

^b^Whole brain radiation was given to 2 patients diagnosed with PCNSL while alive (24 Gy in 8 fractions to one patient and 30 Gy in 10 fractions to one).

^c^Only 2 patients had favourable clinical and radiological response.

**Figure 2:**
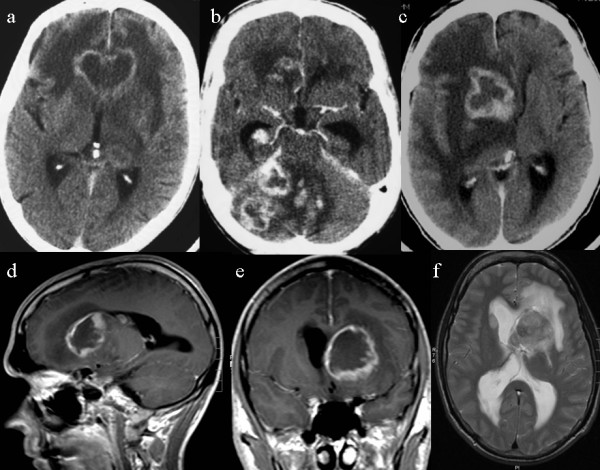
**AIDS-related PCNSL in Norway: imaging findings at CT and MRI**. Contrast enhanced CT in 3 patients demonstrating lesions with ring-enhancement in the frontal lobes (a), cerebellum (b) and basal ganglia (c). MRI (d-f) in another patient with lesion in the basal ganglia at T1 (d-e) and T2 (f) weighted images.

Review of CT/MRI in 14 patients revealed no brain lesion in 1 (7%), a single lesion in 2 (14%), and multiple (2–7) lesions in 11 (79%) patients. In the patient with no lesion, CT one week prior to death indicated hydrocephalus. All together, 37 lesions (36 at CT and 9 at MRI) were identified; involving the lobes in 11 patients (79%) and the deep structures of the brain in 8 patients (57%) (Table 1). All lesions enhanced after intravenous contrast injection; 75% showed ring-enhancement both at CT (27/36) and MRI (6/8) (Figure 2).

The two patients diagnosed while alive received whole brain radiation; no patients received cancer chemotherapy. HAART was given to 4/7 (57%) diagnosed after the introduction of HAART in Norway (August 1996). In 3 patients HAART was started mean (median, range) 4.3 (3.0, 2–8) months prior to first symptom of PCNSL and was continued after symptom debut. Only one of these had a favourable clinical and haematological response, with normalization of CD4 cell count; this response was first achieved after PCNSL was diagnosed. In one patient HAART was started after PCNSL was diagnosed and gave no favourable response. Toxoplasma therapy was given to 20/29 (69%) patients; 2 responded with clinical improvement and regress of imaging findings but both died rapidly after their symptoms relapsed 1 and 15 months after initial toxoplasma therapy, respectively.

**Figure 3:**
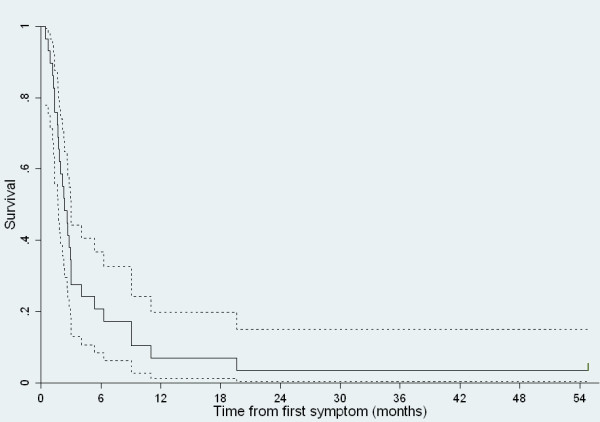
**Survival curve for patients with AIDS-related PCNSL in Norway**. Kaplan-Meier survival curve with 95% confidence interval for patients with AIDS-related PCNSL (n = 29). Median survival from first symptom attributable to PCNSL was 2.3 (95% CI: 1.5–3.2) months.

Median survival from first symptom attributable to PCNSL was 2.3 (95% CI: 1.5–3.2) months. Only 21% (6/29) and 7% (2/29) were alive at 6 months and 1 year, respectively (Figure 3), but one patient has survived for 4.5 years. In this patient PCNSL was established by biopsy 3 months after debut of personality changes; he received HAART and radiotherapy and was alive > 4 years after completed radiotherapy. All deaths (n = 28) were caused by AIDS and PCNSL. Autopsy revealed cerebral toxoplasmosis in addition to PCNSL in 1 patient (not responding to toxoplasma therapy).

## Discussion

Different from non AIDS-related PCSNL in Norway [18], the incidence of AIDS-related PCNSL in Norway decreased in 1989–2003. Previous single- and multicenter studies [8-12] have also found decreasing incidence of AIDS-related PCNSL in the era of HAART. However, to our knowledge, this is the first national survey to confirm these findings.

Our survey had both strengths and limitations. It was based on case reporting to a national cancer registry and a comprehensive search for non-reported cases in the autopsy registry at the hospital treating most AIDS patients in Norway. Furthermore, all cases were histologically verified. However, due to the small sample size and the very few patients diagnosed and treated while alive, we could not assess prognostic factors. Also, most radiological images from 1989–1993 were unavailable. The imaging results should still be quite representative for the years 1994–2003 since images were reviewed for 76% (13/17) of the patients from this time period.

Our figures for incidence rate (1.7 per 100 person-years) and lifetime risk (5.5%) of PCNSL among AIDS patients are in the upper range of figures in other studies [1,6,9,15,20]. Lower incidence rates were reported from the US [9,15]: the absolute incidence rate of brain lymphoma per 1000 person-years among AIDS patients was 4.7 in 1981–1990 [15] and 8.4 and 1.1 in 1988–1995 and 1996–2000 [9], respectively. Although not truly comparable with rates among AIDS patients, reported incidence rates of PCNSL per 1000 person-years among HIV-infected individuals in Europe were 0.3–5.3 in 1983–2002 [11] and 0.4–8.3 in 1994–2000 [10].

Autopsy in all AIDS patients is needed to truly estimate the incidence of AIDS-related PCNSL. In our study, 93% (27/29) had the diagnosis of PCNSL first established by autopsy. None of these patients had a biopsy performed while alive, and 15% (4/27) of them had no focal brain lesion on imaging (Table 1), so PCNSL was unexpected. Furthermore, PCNSL was considered a likely differential diagnosis prior to death in only 35% (8/23) of those with focal brain lesions, whereas cerebral toxoplasmosis was considered likely in 91% (21/23).

The short median survival from first symptom attributable to PCNSL (2.3 months) was similar to survival times in other larger materials (1.0–2.8 months) [8,16,17]. However, lack of a prompt diagnosis of PCNSL may have excluded patients with very poor performance status from optimal treatment. Long time survival of more than 4.5 years was achieved in one patient treated with radiotherapy and HAART. Similar cases have been reported by others [7,21].

Good performance status, higher CD4 count, whole brain irradiation and HAART have favourable impact on survival in AIDS-related PCNSL [1,8,22,23]. Early diagnosis, to ensure optimal treatment before the performance status has declined, is thus essential. In our study, all but 4 of the 27 patients first diagnosed at autopsy had focal brain lesion(s) on CT/MRI, which should raise the suspicion of PCNSL. Furthermore, 17 of 19 patients with focal lesion(s) who received toxoplasma therapy, showed no clear response. None of these 17 patients underwent stereotactic or open biopsy which may be partly explained by the bad performance status (WHO 2–4) in most of them (77%, 13/17).

Delayed diagnosis of PCNSL is a concern with empiric toxoplasma therapy [1,22]. To reduce this delay, brain biopsy is advised when there is clinical or radiological deterioration during the first week of initial toxoplasma therapy or lack of clinical improvement within 2 weeks [1,24]. Also, PCNSL can be diagnosed without awaiting toxoplasma therapy or biopsy, based on CSF Epstein-Barr virus DNA and ^201^Tl single-photon emission computed tomography (SPECT). Combining these two methods, a rapid, accurate and minimally invasive diagnosis may be achievable, although the accuracy of this diagnostic approach is not fully clarified in the era of HAART [2].

The present MRI/CT findings are similar to those in toxoplasmosis [1,25]. MRI and CT can seldom differentiate lymphoma from toxoplasmosis [1,20]. If available, diffusion or perfusion MRI, magnetic resonance spectroscopy, positron emission tomography and SPECT [1,25] might help to select patients for biopsy versus empiric toxoplasma therapy. So might PCR examination of the CSF to detect Ebstein-Barr virus DNA or Toxoplasma gondii DNA [1,2], but none of these tests are completely specific. Biopsy is often needed, but should be weighted against its relatively high procedural risk in AIDS patients [1]. Further research on the combination of less invasive diagnostic procedures is required to reduce the need for biopsy.

## Conclusion

This national survey showed decreasing incidence rate of AIDS-related PCNSL in Norway during 1989–2003. AIDS patients in Norway had a 5.5% lifetime risk of PCNSL, emphasizing the importance of PCNSL as organ-specific manifestation of AIDS. Despite dismal survival in most patients (median 2.3 months), the possibility of long term survival should prompt more aggressive diagnostics in suspected PCNSL.

## Competing interests

The authors declare that they have no competing interests.

## Authors' contributions

ISH, OM and AE conceived of the study and obtained ethical approvals. ISH collected the data, performed the data analyses and drafted the manuscript. JK and ISH reviewed the imaging material. AKG reviewed the histopathological material. OM, AE and OD contributed in the interpretation of data. All authors have critically revised the manuscript and have given final approval of the version to be published.

## Pre-publication history

The pre-publication history for this paper can be accessed here:


